# Complete chloroplast genome of the grain *Chenopodium quinoa* Willd., an important economical and dietary plant

**DOI:** 10.1080/23802359.2020.1845107

**Published:** 2021-01-06

**Authors:** Ming-Ze Gao, Yan-Hui Dong, Virginia Valcárcel, Zhu-Mei Ren, Ya-Li Li

**Affiliations:** a School of Life Science, Shanxi University, Taiyuan, China; b College of Sciences, Shanxi Agricultural University, Taiyuan, China; c Department of Biology (Botany), Faculty of Science, Universidad Autónoma de Madrid, Madrid, Spain; d Department of Biological Science and Technology, Jinzhong University, Jinzhong, China

**Keywords:** *Chenopodium quinoa* Willd., Chenopodiaceae, chloroplast genome, phylogeny

## Abstract

The grain *Chenopodium quinoa* Willd. is the main traditional food of Inca aboriginal, which was a native grain in South American Andes Mountains, the edible and cultivation history of which has been more than five thousand years. In this study, we sequenced the complete chloroplast genome of *C. quinoa* on the Illumina platform by shotgun genome skimming method. The complete chloroplast genome of *C. quinoa* was 152,087 bp in length with the GC content 37.2%, which was comprised of a large single copy (LSC) region of 83,570 bp, a small single copy (SSC) region of 18,107 bp, and a pair of inverted repeats (IRA/IRB) of 25,205 bp. The chloroplast genome encoded 133 genes including 88 protein-coding genes, 37 tRNA genes and eight rRNA genes. Phylogenetic analysis constructed using the maximum likelihood (ML) method strongly supported the monophyly of each genus in the family Chenopodiaceae, and the genus *Chenopodium* is sister to *Spinacia* as a cluster, which closely grouped to *Dysphania.*

The grain *Chenopodium quinoa* Willd., belonging to the family Chenopodiaceae, is a kind of annual tetraploid dicotyledonous herb and also a facultative halophyte, which originated mainly in Bolivia and Peru in South America (Wang et al. 2017; Yang et al. 2019). The species contains a large amount of manganese, zinc, magnesium, calcium and other minerals as well as high-quality complete protein (Hong et al. 2017). Moreover, it contains eight essential amino acids, that cannot be synthesized by the human body but can be widely used in food, daily chemicals, agriculture, and medicine industries (Yao et al. 2019). Because of its unique nutritional value and health care function, *C. quinoa* has attracted international attention and is widely spread in China. At the end of the 20th century, the first trial cultivation began in Tibet, China, and now it has expanded to Gansu, Shanxi, Qinghai, Shaanxi, Yunnan and other places (Gao and Cai 2019). Here, we sequenced the complete chloroplast genome of *C. quinoa* in order to better understand its genomic structure and organization, and analyze its phylogenetic relationship with relative species to provide more useful data for its original and evolutionary research

We collected the sample of *C. quinoa* in the Quinoa Breeding Base of Shanxi Academy of Agricultural Sciences in Jingle County, Shanxi, China (38°54'1"N, 111°22'12"E) in October 2019. The specimen is stored at the Herbarium of School of Life Science, Shanxi University, China (voucher no. SXU-Ren_Krm). We extracted the genomic DNA from the leaves and obtained the chloroplast sequence using the shotgun genome-skimming method on an Illumina HiSeq 4000 platform (Zimmer and Wen 2015). The complete chloroplast genome was assembled using GetOrganelle program (Jin et al. 2018), annotated using DOGMA (Wyman et al. 2004) and Geneious (version 11.0.3; https://www.geneious.com), and then was submitted to GenBank with the Accession No. MT906655. We also deposited the raw sequencing reads in SRA with Accession No. SRR12658560.

The complete chloroplast genome of *C. quinoa* was 1,52,087 bp in length and consisted of four distinct parts: a large single copy (LSC) region of 83,570 bp, a small single copy (SSC) region of 18,107 bp, and a pair of inverted repeats (IRA/IRB) of 25,205 bp. The LSC region contained 61 protein-coding genes and 22 tRNAs, whereas the SSC region contained 11 protein-coding genes and one tRNA. Eight protein-coding genes, seven tRNAs and four rRNAs were duplicated in the IR region. The chloroplast genome totally encoded 133 genes including 88 protein-coding genes, 37 tRNA genes and eight rRNA genes. The overall nucleotide composition is 31.2% A, 19.0% C, 18.2% G and 31.6% T with the total GC content 37.2%.

We combined the protein-coding genes of *C. quinoa* as well as 15 Chenopodiaceae species from GenBank with the two species *Amaranthus caudatus* and *A. tricolor* from the family Amaranthaceae as outgroups to construct the phylogenetic relationship using RAxML program version 8 under GTRGAMMA model with 1000 bootstrap replicates (Stamatakis 2014). The results (Figure 1) strongly supported the monophyly of each genus, and *Chenopodium* is sister to *Spinacia*, which group is closely related to *Dysphania*. The complete chloroplast genome of *C. quinoa* would provide valuable genetic information for further research on its genetic variation and evolution.

**Figure 1.**
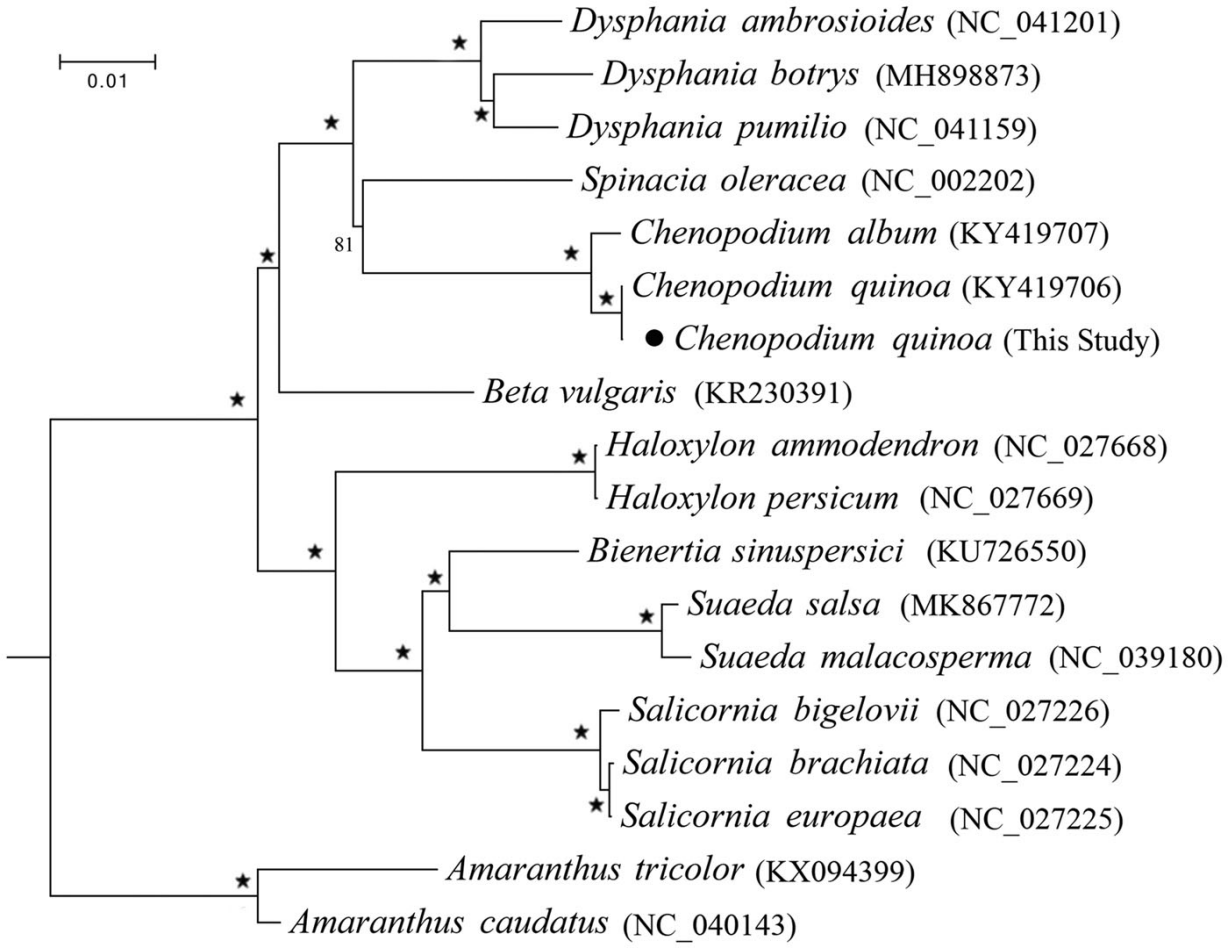
Maximum likelihood (ML) phylogeny of Chenopodiaceae based on all the chloroplast protein-coding genes of 15 species from eight genus with *Amaranthus caudatus* and *Amaranthus tricolor* from the family Amaranthaceae as outgroups. ‘★’ at each node represents the nodes with 100% BS (bootstrap support values).

## Data Availability

The data that support the findings of this study are openly available in GenBank of NCBI at https://www.ncbi.nlm.nih.gov, reference number MT906655. Raw sequencing reads are deposited in SRA of NCBI with the Accession No. SRR12658560.
